# Effects of Propranolol on Neurodevelopmental Outcomes in Patients with Infantile Hemangioma: A Case-Control Study

**DOI:** 10.1155/2018/5821369

**Published:** 2018-02-25

**Authors:** Chuan Wang, Qi Wang, Bo Xiang, Siyuan Chen, Fei Xiong, Yi Ji

**Affiliations:** ^1^Division of Oncology, Department of Pediatric Surgery, West China Hospital of Sichuan University, Chengdu 610041, China; ^2^Pediatric Intensive Care Unit, Department of Critical Care Medicine, West China Hospital of Sichuan University, Chengdu 610041, China; ^3^Department of Child Health, West China Second University Hospital, Sichuan University, Chengdu 610041, China

## Abstract

**Background:**

The aim of this study was to examine whether oral propranolol has any effect on neurodevelopment outcomes in young children with problematic infantile hemangiomas (IHs).

**Methods:**

Thirty-six children with a diagnosis of problematic IH who were treated with oral propranolol were compared with 34 healthy children with no history of propranolol therapy. Patients received propranolol therapy for at least 3 months. Gesell developmental schedules (GDS) were used to evaluate neurodevelopment outcomes in the two groups. The scores of each GDS domain were compared between the two groups.

**Results:**

There were no significant differences in developmental quotient (DQ) values for any of the five domains between the patients and healthy controls (*P* < 0.05). Multiple stepwise regression analyses showed that none of the domains in the control group were influenced by the children's gender or age (*P* < 0.05). In addition, we found that gender, age at the initiation of therapy, age at the time of the neurodevelopment test, and treatment duration had no effect on any domain of the GDS in the patient group (*P* > 0.05).

**Conclusion:**

Propranolol has no obvious effect on neurodevelopmental outcomes in children. Early treatment and treatment duration had no negative effect on central nervous system (CNS) development.

## 1. Introduction

Infantile hemangioma (IH) is the most common benign tumor in children. Most IHs are small, do not cause any complications, and involute naturally without treatment. However, approximately 10–15% of IHs can lead to compromised function and permanent disfigurement or are life-threatening. In certain cases, timely intervention is crucial for preventing potential sequelae [1, 2]. Currently, propranolol is the first-line treatment for IHs due to its excellent efficacy [3]. However, adverse events are common, including sleep disturbance, agitation, acrocyanosis, and gastrointestinal symptoms [4]. Some serious adverse events, including symptomatic hypotension, hypoglycemia, and bradycardia, have also been reported [3].

Propranolol is a lipophilic nonselective *β*-adrenergic receptor (*β*-AR) blocker and has the ability to easily penetrate the blood-brain barrier [5]. It is well established that noradrenaline neurotransmitters play an important role in the early development of the central nervous system (CNS) [6]. Both agonists and antagonists of *β*-ARs act antithetically via the same intracellular pathways [7]. The use of propranolol in the treatment of IHs is usually initiated in infancy, which is a critical time for CNS development. These issues raise questions regarding the assumption that propranolol may have the capacity to exert effects on CNS development in young infants [8].

Interestingly, although clinical data in children are rare, evidence for the effects of propranolol on CNS function in adults is plentiful, albeit controversial [8]. In this regard, studies demonstrated that propranolol could impair memory, psychomotor functions, and mood in adults [6, 8]. Mihov et al. found that propranolol had an obvious effect on the facilitation of declarative learning by social-emotional feedback [9]. Remarkably, sleep disturbance is one of the most common adverse effects of propranolol in infants [10]. There is direct evidence that acute sleep restriction has a negative impact on emotion responses in children [11].

Unfortunately, although propranolol is widely used to treat IHs, little is known regarding the impact of propranolol on CNS development in children. Clinically, the initiation of propranolol treatment for cosmetic IHs is a difficult choice to make. Therefore, we conducted this study to assess the impact of propranolol on CNS function in young children by using Gesell developmental schedules (GDS) and identifying related risk factors of CNS function (if they exist), with the aim of providing meaningful data regarding the safety of propranolol therapy in children.

## 2. Methods

This study was approved by the Ethics Committee of West China Hospital of Sichuan University and West China Second University Hospital of Sichuan University. All procedures followed the research protocols that were approved by West China Hospital of Sichuan University and West China Second University Hospital of Sichuan University and were conducted according to the Declaration of Helsinki.

### 2.1. Patients and Health Children

All patients with IH were recruited at the Department of Pediatric Surgery, West China Hospital of Sichuan University, from January 2015 to December 2015. The inclusion criteria were as follows: (1) patients were aged <12 months; (2) patients had received propranolol therapy due to problematic IHs, and the duration of treatment was ≥3 months; and (3) patients did not have any known risk factor for developmental delay or growth restriction. The exclusion criteria were the following: (1) patients had received or were currently receiving other treatments or any comedication for IHs; (2) the duration of propranolol treatment was <3 months; and (3) patients have PHACE syndrome (posterior fossa malformations, hemangiomas, arterial malformations, coarctation of the aorta and other cardiac defects, and eye anomalies). Age- and gender-matched control subjects were healthy children. They were recruited from the Department of Child Health, West China Second University Hospital of Sichuan University. Written informed consent was obtained from each child's parents.

### 2.2. Propranolol Treatment

The patients' parents were informed that propranolol was prescribed for the treatment of IH, and they provided written, informed consent for treatment. Propranolol was initiated at a dose of 1.0 mg/kg/day divided 3 times daily for 1 week, and the dose was then increased to 2.0 mg/kg/day in 3 daily doses beginning in week 2. The dosage of propranolol was adjusted for weight (2 mg/kg per day). The duration of propranolol use was noted.

### 2.3. Instruments

GDS, developed by Gesell, were designed to evaluate broad CNS development. This instrument is widely used due to its excellent reliability and validity [12]. GDS provided a neurodevelopmental profile in five domains (adaptive, gross motor, fine motor, language, and personal/social) [12]. The neurodevelopmental status was measured by the development quotient (DQ) of the five domains. The DQ was derived by dividing the age-equivalent level of each domain according to the child's chronological age at the time of the neurodevelopmental assessment [13]. A higher DQ indicates better neurodevelopment. Considering that prematurity is an important risk factor for IH [14], GDS may be better for the assessment of neurodevelopmental status than other instruments. GDS was conducted by specially trained pediatric physicians and nurses in West China Second University Hospital of Sichuan University.

### 2.4. Statistical Analysis

All analyses were conducted by using SPSS 22.0 for Windows (SPSS Inc., Chicago, IL, USA). An independent samples *t*-test was used to analyze the continuous variables, and chi-square (*χ*2) tests were used to compare qualitative variables. Multiple stepwise regression analyses were used to identify the main factors influencing GDS. Statistical significance was set at *P* < 0.05.

## 3. Results

Thirty-six patients with IHs were recruited in this study. And thirty-four healthy children with a mean age of 12.59 months were enrolled in the healthy control group. Demographic characteristics of two groups were listed in Table 1. There were no significant differences in demographic characteristics between the two groups (*P* > 0.05).

DQ values of each domain in the two groups are shown in Table 2. We found no significant difference between corresponding DQ values in the five domains in the two groups (*P* > 0.05).

The outcomes of multiple stepwise regression analyses of DQ values of each domain in the healthy control group and the patient group are listed in Tables 3 and 4, respectively. In the control group, age and gender did not have a significant effect on DQ values in any of the GDS domains (*P* > 0.05). In the patient group, we found that gender, age at the initiation of therapy, age at the time of the neurodevelopment test, and treatment duration had no effect on any domain of the GDS (*P* > 0.05).

## 4. Discussion

Propranolol has been widely used to treat IHs since Léauté-labrèze et al. serendipitously discovered the dramatic effects of propranolol in inducing an accelerated involution of problematic IHs [15]. However, although propranolol is efficacious, there are theoretical concerns regarding the potential significant neurodevelopmental or cognitive side effects of propranolol in children [16].

Noradrenaline neurotransmitters in the CNS are mainly released from the locus coeruleus and are strongly associated with the functions of the amygdaloid nucleus and the hippocampus [17], both of which are involved in emotion experience [18–20]. The high liposolubility of propranolol can facilitate the passage of propranolol across the blood-brain barrier. Thus, propranolol has been used to relieve anxiety symptoms and prevent posttraumatic stress disorder [21, 22]. Stoschitzky et al. demonstrated that propranolol was able to block the 5-hydroxytryptamine receptor, resulting in decreased production of melatonin and influencing CNS function [23]. Clinically, children who receive propranolol therapy may be at a higher risk of CNS-related adverse events (e.g., sleep disturbance, agitation, and somnolence) [10]. Therefore, whether propranolol has a negative impact on CNS function in children is a significant concern. Clinicians may face a dilemma in determining whether to treat IHs with propranolol for a cosmetic reason.

The accurate measurement of CNS function in normal children is sometimes difficult. GDS could be used to broadly assess the neurodevelopmental status in children; additionally, they provide a reliable measure of development in this age group and yield separate scores for mental and motor development [24, 25]. In the present study, we used GDS to determine that propranolol therapy had no negative impact on neurodevelopmental outcomes in young children with IHs.

Our study showed that oral propranolol at a dose of 2.0 mg/kg/day did not impair early fine or gross motor development in young children with IHs. These findings were consistent with the serial studies by Moyakine et al., which demonstrated that propranolol neither negatively influenced fine or gross motor function nor affected psychomotor development in patients with IH [26–28]. Remarkably, our study provides further evidence that propranolol therapy for a mean duration of 9.40 months did not impair adaption and personal social function in young children. Notably, the age at the initiation of propranolol treatment in our patients was in the first year of life, and this period is the most sensitive period for the development of language function [29]. We noted evidence for the equivalence between patients treated with propranolol and healthy controls in terms of language function domain. Our study provided valuable results indicating that propranolol had no negative impact on the development of language function.

As mentioned above, propranolol has an impact on the functions of the amygdaloid nucleus and the hippocampus. These two domains are also involved in memory function [30]. Therefore, concern rises continuously regarding the possible effects of propranolol on memory function. Cahill and Van Stegeren substantiated that the use of propranolol for a short time had a negative impact on the memory in adult [31]. However, CNS has a strong plasticity in infancy. Theoretically, alternative pathway may compensate the memory function for a long-term use of propranolol. Unfortunately, previous studies failed in clarifying the puzzle [10]. In our study, the GDS is also unable to test the impact of propranolol on memory function. Therefore, question still exists as whether long-term use of propranolol has a negative effect on memory function in infancy. Further studies are needed to elucidate this question.

Recently, studies have revealed that the rebound or relapse of IHs occurred more frequently in patients who completed propranolol treatment before 9 months of life [32]. Usually, the duration of propranolol treatment was longer than 6 months [33]. In the present study, the mean duration of propranolol therapy was 9.40 months. Thus, the majority of our patients received propranolol treatment with a duration that may cover the entire proliferative phase. Although several studies have shown that propranolol had a significantly negative impact on CNS function, the duration of oral propranolol was short [9, 30, 34]. Otherwise, it is conceivable that CNS function was influenced by propranolol in the short term, but this function may be compensated by brain plasticity during long-term treatment.

We found that the age at the initiation of therapy had no effect on CNS development. This finding is exciting and suggests that the CNS risks associated with the use of propranolol in young infants may be lower than we initially feared. Previously, Chang et al. demonstrated that the largest increase in IH tumor size occurred in the first 3 months of life, and by 5 months of age, both segmental and localized IHs had reached 80% of their final size [35]. Some IHs are associated with a high risk of functional or cosmetically critical changes even in the early proliferative phase. Although propranolol treatment is effective in both arresting growth and causing involution, irreversible sequelae may have already occurred. These observations, together with the work presented here, promote the recommendation that, in patients with severe IHs, oral propranolol should be administered as early as possible to avoid potential complications [36, 37].

The limitations of this study include its resultant small cohort sizes. In addition, some CNS functions cannot be accurately measured until the age of 6-7 years. There is insufficient data, at our institutions and in the literature, in reporting long-term CNS function in order to ensure the safety of propranolol in the treatment of IHs.

## 5. Conclusion

In conclusion, we demonstrated satisfactory neurodevelopmental outcomes in IH patients treated with propranolol. Although long-term follow-up data are lacking, our current data demonstrated that propranolol does not produce neurodevelopmental delays in young children for short-term use. In addition, we revealed that the duration of propranolol therapy did not increase neurodevelopmental risk. Early treatment with propranolol had no negative effect on CNS function.

## Figures and Tables

**Table 1:** Demographic characteristics of the patient group and the healthy group.

Variable	Patient group	Control group	*P* values
Gender^*∗*^			0.663
Male	13 (36.1)	14 (41.2)	
Female	23 (63.9)	20 (58.8)	
Gestational age^*∗*^			
Term born (≥37 weeks)	30 (83.3)	29 (85.3)	0.822
Born prematurely (<37 weeks)	6 (16.7)	5 (14.7)	
Age at neurodevelopment test (m)	12.27 ± 1.56	12.59 ± 2.06	0.478

^*∗*^Values are presented as the number (percentage). Values are presented as the mean ± standard deviation.

**Table 2:** Scores of each domain in the patient group and the healthy group^*∗*^.

Domain of GDS	Patient group (mean ± SD)	Control group (mean ± SD)	*P* values
Adaption	94.81 ± 10.01	96.57 ± 16.21	0.584
Gross motor	98.20 ± 11.41	95.39 ± 9.40	0.365
Fine motor	101.98 ± 11.81	102.20 ± 15.28	0.945
Language functioning	88.53 ± 12.05	88.01 ± 15.13	0.872
Personal social functioning	98.11 ± 17.48	92.38 ± 9.19	0.940

^*∗*^GSD: Gesell developmental schedules; SD: standard deviation.

**Table 3:** Factors influencing the scores of each domain in the healthy group^*∗*^.

Variable	Gender		Age	
	*β* values (95% CI)	*P* values	*β* values (95% CI)	*P* values
Adaption	−0.008 (−10.703–10.263)	0.966	0.144 (−1.531–3.549)	0.424
Gross motor	−0.124 (−8.866–4.212)	0.473	0.301 (−0.214–2.956)	0.087
Fine motor	−0.161 (−15.967–6.092)	0.368	−0.082 (−3.279–2.066)	0.647
Language functioning	−0.075 (−13.198–8.651)	0.674	−0.162 (−3.834–1.460)	0.367
Personal social functioning	−0.179 (−9.862–3.268)	0.314	−0.139 (−2.210–0.971)	0.443

^*∗*^Multiple stepwise regression analyses were used to determine the main risk factors; CI: confidence interval.

**Table 4:** Factors influencing the scores of each domain in the patient group^*∗*^.

Variable	Adaption		Gross motor		Fine motor		Language functioning		Personal social functioning	
	*β* values (95% CI)	*P*	*β* values (95% CI)	*P*	*β* values (95% CI)	*P*	*β* values (95% CI)	*P*	*β* values (95% CI)	*P*
Gender	0.058 (−5.812–8.184)	0.732	−0.119 (−11.689–6.091)	0.526	0.088 (−6.722–10.976)	0.627	−0.109 (−11.332–5.950)	0.530	0.101 (−9.666–16.939)	0.581
Age at initiation of therapy	0.154 (−18.479–20.308)	0.924	0.397 (−21.955–27.319)	0.826	−0.712 (−29.5031–19.544)	0.682	1.884 (−10.508–37.386)	0.261	2.644 (−9.515–64.214)	0.140
Age at neurodevelopment test	−0.653 (−23.404–15.031)	0.660	−0.586 (−28.696–20.130)	0.723	0.255 (−22.373–26.228)	0.873	−1.516 (−35.439–12.020)	0.322	−2.218 (−61.368–11.690)	0.175
The duration of treatment	0.241 (−18.132–20.549)	0.899	0.399 (−22.291–26.848)	0.851	−0.718 (−28.707–20.205)	0.725	−2.445 (−9.113–38.649)	0.217	3.144 (−9.666–16.939)	0.581

^*∗*^Multiple stepwise regression analyses were used to identify the main risk factors; CI: confidence interval.
